# Implementing Lung Cancer Screening and Prevention in Academic Centers, Affiliated Network Offices and Collaborating Care Sites

**DOI:** 10.3390/jcm9061820

**Published:** 2020-06-11

**Authors:** Cary A. Presant, Ravi Salgia, Prakash Kulkarni, Brian L. Tiep, Shamel Sanani, Benjamin Leach, Kimlin Ashing, Jossie Sandoval, Mina S. Sedrak, Shana Landau, Sophia Yeung, Dan Raz, Shanmugga Subbiah

**Affiliations:** 1 City of Hope Medical Center, Duarte, CA 91010, USA; rsalgia@coh.org (R.S.); pkulkarni@coh.org (P.K.); btiep@coh.org (B.L.T.); ssanani@coh.org (S.S.); bleach@coh.org (B.L.); kashing@coh.org (K.A.); asandoval@coh.org (J.S.); msedrak@coh.org (M.S.S.); slandau@coh.org (S.L.); syeung@coh.org (S.Y.); draz@coh.org (D.R.); ssubbiah@coh.org (S.S.); 2 City of Hope Medical Center, West Covina, CA 91790, USA

**Keywords:** cancer center, lung cancer, lung cancer screening, low-dose CT scans, cancer prevention, smoking cessation, tobacco control, national guidelines for screening and prevention, pharmaceutical aids to smoking cessation

## Abstract

Lung cancer is one of the deadliest and yet largely preventable neoplasms. Smoking cessation and lung cancer screening are effective yet underutilized lung cancer interventions. City of Hope Medical Center, a National Cancer Institute (NCI)- designated comprehensive cancer center, has 27 community cancer centers and has prioritized tobacco control and lung cancer screening throughout its network. Despite challenges, we are implementing and monitoring the City of Hope Tobacco Control Initiative including (1) a Planning and Implementation Committee; (2) integration of IT, e.g., medical records and clinician notification/prompts to facilitate screening, cessation referral, and digital health, e.g., telehealth and social media; (3) clinician training and endorsing national guidelines; (4) providing clinical champions at all sites for site leadership; (5) Coverage and Payment reform and aids to facilitate patient access and reduce cost barriers; (6) increasing tobacco exposure screening for all patients; (7) smoking cessation intervention and evaluation—patient-centered recommendations for smoking cessation for all current and recent quitters along with including QuitLine referral for current smokers and smoking care-givers; and (8) establishing a Tobacco Registry for advancing science and discoveries including team science for basic, translation and clinical studies. These strategies are intended to inform screening, prevention and treatment research and patient-centered care.

## 1. Introduction

Lung cancer is the leading cause of cancer-related deaths. However, lung cancer is one of the most preventable human malignancies. A screening program is able to detect lung cancer at an earlier and more successfully treatable stage, and thus improves survival. In order to control lung cancer incidence, morbidity and mortality, it is important for a health care delivery system to provide and monitor screening and prevention programs including tobacco cessation. In this report, we will review the experience of the City of Hope Medical Center at its academic campus in Duarte, CA, as well as its 27 community practice sites throughout much of Southern California. This is an observational descriptive study, and broader results of our experience are under study and will be published separately. 

### 1.1. City of Hope’s Experience in Lung Cancer Screening and Prevention

**Figure 1:**
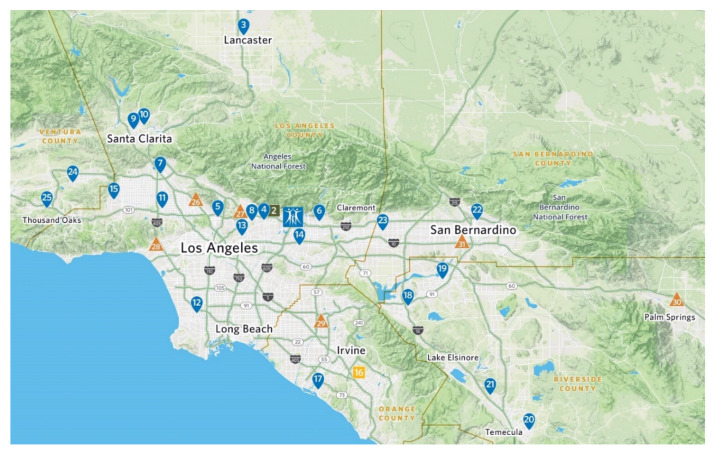
Map of City of Hope Locations in Southern California. Blue Square, Duarte National Medical Center Academic Site; Blue Teardrops, Community Cancer Center Practice Sites; Orange Triangles, City of Hope Oncologist-Staffed Community Hospitals; Yellow Square, Orange County Campus (under construction). Map designed at City of Hope Medical Center.

City of Hope is an NCI-designated comprehensive cancer center and a member of the National Comprehensive Cancer Network (NCCN) network. City of Hope utilizes the NCCN guidelines for lung cancer screening and prevention. In addition, all clinical sites (the academic center in Duarte and all 27 community centers) follow the NCCN lung cancer care and screening guidelines and comply with Via Oncology Pathways (as modified by City of Hope) for evaluation, anti-tumor treatment and surveillance after treatments. A map of these sites indicates the broad coverage of communities in the greater Los Angeles area (Figure 1). Compliance with these pathways is monitored for every physician and advanced practice practitioner (APP). Pathway compliance is very strongly encouraged. We follow the NCCN guidelines on smoking cessation, since smoking cessation is conceptually an integral component of cancer care.

**Figure 2:**
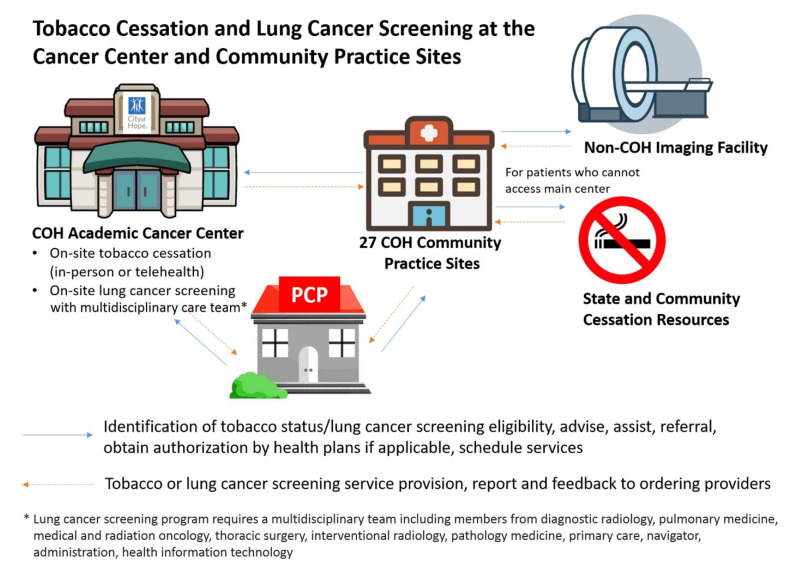
Lung Cancer Prevention and Screening Model. The City of Hope Lung Cancer Tobacco Control and Cancer Screening Initiative Is Implemented in the above Model. Model was designed by City of Hope Medical Center.

Lung cancer team leadership at City of Hope is provided by Dr. Ravi Salgia, Dr. Dan Raz and Dr. Erminia Massarelli. Under their direction as champions, the lung cancer disease team promotes lung cancer screening and prevention in Duarte and in all network community centers. The relationships between these components of the program are pictured in Figure 2. This model following the team leadership with advice of the Department of Population Science allows interactive design of the initiative and modifications, and implementation in Duarte and community sites and a synergistic effect implementing screening and prevention. 

### 1.2. Academic Center Experience

At the academic center, City of Hope conducts preclinical laboratory investigations into genetic factors associated with lung cancer. This research helps to define which patients are at risk for developing lung cancer. Genomic alterations are identified which may help in screening and development of drugs targeted for cancer prevention. Population science research further aids in cancer control activities. Clinical trials, developed from these research activities, are initiated at the academic campus in Duarte as well as at one or more of our 27 affiliated network community cancer centers.

At the academic center, cancer program members (67 physicians, 67 APPs, 12 research faculty, 14 graduate students and fellows and 10 additional data analysts, and clinical trial coordinators) and specifically the lung cancer team conduct weekly disease team-based meetings/tumor boards/research seminars and invite participation of physicians at the network community cancer center offices. At the academic center, screening with low-dose CT (LDCT), smoking cessation, and patient education are provided.

At the academic center, 8623 total new patients including 3255 new cancer patients are seen annually (data from 2019), with 252 new lung cancer patients evaluated per year. Racial/ethnic diversity exists, with 61.4% Caucasian, 19.9% Hispanic, 14.7% Asian/Pacific Islander, and 3.9% African-American patients. Overall, 4.2% of all patients at the academic center self-report as current smokers, 27.8% are former smokers, and 61% of patients are never smokers. Medicare is the payer in 37.9%, Medicare Advantage in 1.7%, Medicaid in 9.3%, PPO or commercial in 29.1%, HMO or managed care in 17.9%, and other payers in 3.4% of patients. This patient population is different compared to the 27 community centers, which has implications for screening and tobacco control. Actual practices in tobacco control vary by treatment site. At the academic center, there is a formal smoking cessation program, while there are no formal smoking cessation resources at network community offices. At all sites, patients are referred to the California Smokers Helpline 800 No Butts program (800-No-Butts) or to the California QuitLine operated out of the University of California San Diego (800 Quit Now).

At the academic center, LDCT screenings were ordered in 434 patients over a 4 year period, and 424 (98%) completed the procedure. We universally use the Lung-RADS system of evaluation of LDCT scans [1]. Of those patients undergoing LDCT, Lung-RADS results showed suspicious lung cancer category 4A results in 3.3% and highly suspicious category 4B results in 1.7%. Of the six patients who had biopsies performed at the academic center, four (67%) had stage 1 lung cancer diagnosed, and two (33%) had stage 4 lung cancer. Other patients were referred back to their primary care physicians for diagnostic biopsies. Long-term follow up of patients diagnosed through our LDCT screening program will determine reductions in risk of death and will be reported in the future. Any cost savings associated with earlier diagnosis depend on the health system, and different health payers are involved with our patients (see above).

One of the issues in lung cancer screening is the excessive use of scans. At City of Hope, Shared Decision-Making Consults and Lung-RADS are some of our tools to address the issue of reducing excessive imaging, false positives, excessive harm as a result of the additional testing, patient anxiety, and cost. During the Shared Decision-Making Consults, ordering clinicians use checklist and visual tools to discuss the risk and benefits with the patients, address patients’ concerns, and assist patients to determine whether the lung cancer screening is suitable. The clinician also educates patients on what is low-dose CT, and advises postponing the screening if they have had a chest/PET-CT in the past 12 months. In the Duarte cancer center LCS program, we also coordinate with the attending clinicians to prevent excessive imaging. Lung-RADS is a widely accepted and logical nodule reporting and management tool used by the radiologists to minimize false-positive findings, excessive imaging, and unnecessary procedures. As part of our lung cancer screening initiative, we are developing educational tools and informational aids to help patients understand these issues before screening.

### 1.3. Community Centers Experience

Our community cancer centers are staffed with 43 medical oncologists, 40 radiation oncologists, 7 APPs and a clinical trials coordinator. In order to improve lung cancer control, physicians and APPs at the 27 network community cancer centers advise screening and prevention at local tumor boards and medical lectures, and depend on collaborating/referring community primary care physicians, pulmonologists, radiologists and hospitals for lung cancer control. Primary care physicians who are the referring doctors of patients to City of Hope, as well as City of Hope providers themselves (to varying degrees), make assessments of lung cancer risk, order LDCT screening exams, advise or provide smoking cessation to current smokers or recently quit patients, refer patients to other local smoking cessation programs if available, prescribe smoking cessation medications, and provide behavior intervention and counseling with follow-up visits to assess compliance. 

In only one-half of the network community practices do the oncologists prescribe medications to assist in smoking cessation, while the other half simply refer the patients back to their primary care physicians with recommendations. In a current survey of community practice sites, almost all of the network community center physicians discuss screening and prevention at tumor boards or hospital meetings where primary physicians and pulmonologists are in attendance, but a few still do not, even today.

At the 27 community centers, 22,000 new patients are seen annually (data from 2019). In total, 19,201 patients are seen by medical oncology/hematology specialists. The incidence of current smokers is 7.2%. Of the patients, 49.4% are Caucasian, 20.3% are Hispanic, 4.5% are African-American, and 6% are Asian/Pacific Islander. Among our patients, 43.7% are insured by government programs, 22.9% are PPO/commercially insured, and 22.7% are insured by HMOs or IPA medical groups.

### 1.4. Institutional Vision

Even in an integrated oncology network, there remain challenges with opportunities for improvement. In our vision, there is a focus on team medicine. The academic center uses its knowledge, skills and experience to set standards and create pathways so as to encourage its network community centers to implement the science and evidence base (Table 1). The community center encourages the local collaborating physicians, medical groups, IPAs, and hospitals to utilize their resources to implement the screening and prevention activities as well as providing smoking cessation advice and medications and/or nicotine replacement treatments for some patients. Naturally, these necessitate that the primary care physician, pulmonologist and/or oncologist order the interventions necessary to reduce the risks of lung cancer. The component activities or interventions seen in Table 1 are most appropriately (indicated by +++) taking place at the academic center or network community cancer center. 

To address these, we have developed the City of Hope Tobacco Control Initiative. Elements of the initiative were begun in 2013. As the program elements matured and new information informed needed improvements and coordination, the initiative was revised. The current initiative was formulated in January 2020. It includes (1) a Planning and Implementation Committee; (2) integration of IT including medical records and clinician notification/prompts to facilitate screening, cessation referral; (3) physician and clinician training and supervision that endorses national guidelines and refer and encourage lung cancer screening and smoking cessation interventions; (4) providing a clinical champion at the community sites, and at the academic center for program leadership; (5) Coverage and Payment reform and aide to facilitate patient access and utilization and reduce cost barriers; (6) implementing systemic tobacco exposure screen for new and existing patients; (7) smoking cessation intervention and evaluation—patient-centered recommendation for smoking cessation for all current and recent quitters along with QuitLine referral for all current smokers and the smoking care-givers; and (8) establishing a Tobacco Registry for advancing science and discoveries including team science for basic, translation and clinical studies.

The implementation of the initiative is in progress at Duarte and in community sites, as led by the lung cancer disease team, clinical departments, and the network, regional and individual site leaders, and community center lung cancer champions. Evaluation methods include a review of electronic health records for LDCT screening orders in patients with higher risk based on smoking history and other clinical and epidemiologic factors, referral for smoking cessation, continuing visits for monitoring cessation efforts, and frequency of prescribing anti-smoking medications and/or nicotine replacement therapy medications. Compliance measures are thus multifactorial. At present, we have demonstrated that all 27 community cancer centers can operate together, in a coordinated well-integrated effort with the academic site.

## 2. Focus on Lung Cancer Screening

### The Science

Unfortunately, LDCT is a lung cancer screening method which is underused [2]. The frequency of performing lung cancer screening in patients who are eligible for screening by nationally accepted criteria is 3.9% as of 2015 [2]. However, this may be improving, since a recent study suggests that in a survey of 10 states in 2017, 12.7% of people 55–80 years old were eligible for screening by national criteria, and 12.5% actually had received screening [3]. There was a variation by state ranging from 9.7% to 16%. 

As summarized in NCCN guidelines [4], patients who are generally eligible for LDCT screening are those at high risk, defined as 55 to 77 years old, have a 30 or more pack year history of smoking, and are active smokers or have stopped smoking within the last 15 years. Although not defined in the screening trials as high risk, other patients who are also at higher risk are those who are age 50 years or over, with a 20 or more pack year smoking history, and have other risk factors. These risk factors can include prior lung or head and neck cancer, a family history of lung cancer, prior chest radiotherapy, asbestos or radon exposure, presence of HIV or COPD or pulmonary fibrosis, or significant second-hand smoke exposure.

Criteria for evaluation are also nationally standardized as outlined in the NCCN guidelines [4]. They cover not only which findings require immediate biopsy or excision, but also which require close follow up versus those who require a repeat LDCT in 1 year.

LDCT has been shown to detect earlier state lung cancer. In fact, 63% of lung cancers which were found by LDCT were stage IA or IB [5]. Further, this earlier detection has been associated with reduced lung cancer deaths by 20% worldwide [6]. 

It is important to identify patient symptoms which might be associated with lung cancer. Persistent cough, hemoptysis, shortness of breath, chest pain, weight loss, and/or wheezing may be symptoms of lung cancer. Someone who has any of these symptoms should not get a low-dose CT scan, but they should get a diagnostic CT scan with and without contrast. Payment for diagnostic CT in the presence of symptoms is covered by insurance even in managed care plans. Requests for prior authorization should include diagnostic codes for the specific symptoms. For patients with prior lung cancer, annual diagnostic CT chest scans are indicated, with details included in NCCN guidelines. Clinicians both at Duarte and in community sites are committed to compliance with those guidelines. 

If the LDCT is negative, patients should be counseled regarding prevention. This is addressed in the following section. Since the prevalence of LDCT screening is low among patients who meet requirements to receive it, there remains considerable need for medical leadership in local communities to encourage more widespread use.

## 3. Smoking Prevention

### The Science

The most prevalent causative factor for lung cancer is smoking—an estimated 85–90% of patients [7]. Thus, smoking cessation is crucial. Smoking cessation programs include a recommendation from a physician to a patient to stop smoking, followed by referral to a smoking cessation program. In order to prevent withdrawal symptoms and facilitate cessation, the physician can prescribe pharmaceuticals such as varenicline, nicotine replacement therapy (NRT), or buproprion with or without NRT [8]. The success of cessation in achieving complete abstinence is poor with a 1 year abstinence rate of 20% using buproprion with NRT, and a 24 week abstinence rate of 26% with varenicline [8]. Behavioral interventions have been shown to improve odds of success over medications alone. Therefore, follow up is most necessary to continue to promote cessation on the individual patient level. Referral to smoking cessation is best performed by the primary care physician or APP, since their relationships with the patient are generally closer than relationships with the oncologists. However, cessation is also often prescribed and facilitated by an oncologist as part of standard cancer care. Further, the oncologist has a role in promoting smoking control at the collaborating hospital or medical staff meetings. The immediate and long-term benefits of smoking cessation include not only better survival, but also critical positive impact on cancer care, improved success of treatment, reduced recurrence of the primary cancer, less frequent development of new cancers, and less progression of comorbid conditions.

If smoking cessation is achieved, lung cancer incidence rate is reduced by 83% in totally abstinent non-smokers, and by 55% in partially abstinent smokers [9]. Recidivism rate, with patients again starting to smoke, are unfortunately high. Continued motivational support for the patient is necessary. Many hospitals including the City of Hope academic center in Duarte have smoking cessation support programs. Alternative programs are provided by such organizations as the American Cancer Society California Division and state of California via phone or internet-based support program called 1-800-No-Butts.

There is high and growing use of E-cigarettes for either nicotine replacement or combustible smoking cessation. The exact impact of E-cigarettes in transitioning from combustible smoking to non-smoking remains controversial. Recent studies cast doubt on the true efficacy of substituting vaping for combustible cigarette smoking. For many individuals who turn to vaping, dual use is common. This leads to an additive effect of toxicities from smoking and vaping, which is counter to the overall goal of tobacco cessation.

City of Hope has helped to develop and adheres to NCCN guidelines on smoking cessation. We do not recommend E-cigarettes or vaping as a smoking cessation or smoking replacement tool either for single use or in combination with FDA-approved smoking cessation medication. If a patient has quit smoking using E-cigarettes, we support continuation of non-smoking status, encourage transition to approved smoking cessation medications, encourage behavior support, and provide support to reduce the risk of lapses or relapses. Of special concerns are the dual use of tobacco and E-cigarettes and E-cigarettes in children where lifetime addiction to nicotine products are of particular concern.

In addition to smoking cessation, other interventions are known to be effective in reducing lung cancer rates and are advised by the National Institutes of Health [10,11]. Patients should avoid second-hand smoke, avoid excessive radiologic scans, reduce exposure to radon (present in 1 of every 15 homes), not be exposed to asbestos (and also chromium, nickel, beryllium, cadmium, tar, uranium, coal products and diesel fuel), and avoid beta carotene use in smokers. 

Other interventions to reduce lung cancer risk which have evidence to support them but not yet accepted in national guidelines include eating a Mediterranean-style diet with increased fruits and vegetables, possibly leading to a 33–50% reduction in lung cancer [12]; increasing exercise, with possibly a 25% reduction [13]; or using green tea, with possibly a 22% reduction [14]. 

A number of nutritional interventions have been recommended without clear evidence of possible improvement. These interventions include red wine, flax seed, garlic, ginger, selenium, turmeric and vitamin D. 

## 4. Translating Screening and Prevention from the Academic Center

It is difficult to successfully promote lung cancer screening and prevention at the local community since insurance often does not adequately reimburse for these programs or for pharmaceuticals. The evidence base is clear that screening and prevention should be strongly encouraged. Our survey of primary care physicians in our catchment area of greater Los Angeles indicated that these physicians were aware of LDCT screening guidelines—only 12% of primary care physicians in the community (not at academic centers) had referred patients for lung cancer screening in the past year [15]. This indicates a need for community leadership by City of Hope oncologists at local hospital meetings and tumor boards to promote appropriate LDCT screening. 

There are many factors that help to promote these programs (Table 2). Guidelines exist for screening and prevention, but they must be more widely accepted by physicians, medical groups, IPAs, HMOs, and hospitals. This must be based on a collaborative relationship, which must be fostered by network community cancer centers and collaborating local physicians and APPs. Promotion can be most effective at tumor boards, at continuing medical education presentations, and even in lunchrooms. Hospitals often need to differentiate themselves from competing institutions, and controlling lung cancer can be an important factor. Having champions in the hospital and on the medical staff can help, and often philanthropy can foster a successful program of smoking cessation. Coordination with voluntary health care organizations can provide educational and patient-support resources not otherwise available in the community.

However, many obstacles exist in successfully controlling lung cancer. Pressure to reduce health care spending is part of the national debate, and motivates lower authorization of screening and prevention efforts at the insurer, IPA, HMO, alternative payment model (APM) and medical group administrative level. Contracts between those organizations and individual physicians and APPs may limit ability to refer for screening and prevention interventions. Even with approval for LDCT screening or smoking cessation, many hospitals do not have resources available. Since it takes time to evaluate lung cancer risk, and order tests and preventive interventions, there is often a lack of reimbursement for those individual patient visits. 

Importantly, compliance surveys on which contracts and payments are based only infrequently assess lung cancer screening and prevention activities. There is poor coordination of patient care in the area of lung cancer screening and prevention. Even when a health plan approves screening or smoking cessation, copayments by patients may still be high for those interventions as well as for medications to suppress withdrawal symptoms. 

Just to reemphasize, physicians and APPs should always distinguish between screening LDCT, versus the need for diagnostic chest CT. A diagnostic CT is appropriate and indicated if patients have any symptoms that could be associated with lung cancer. Ordering the appropriate test is most important. 

Another opportunity for enhancing local community hospital implementation of lung cancer screening is the evolution of algorithms for interpreting LDCT scans. As software and analytics improves, we expect to see new criteria for identifying lung nodules likely to be neoplastic. Although sometimes difficult for a local community radiologist to remain current in updates, the City of Hope Department of Radiology can provide educational updates for local community hospital radiologists to be immediately aware of the new algorithms necessary for earliest diagnosis of lung cancers more amenable to curative interventions. 

## 5. Recommendations for Translating Screening and Prevention into Local Medical Communities

Although there are obstacles for lung cancer control, advances in the science of lung cancer screening, prevention and treatment can favorably impact cancer care. The success of the City of Hope programs across the entire enterprise of academic center and 27 affiliated network community sites has led us to be able to recommend steps that other institutions can take to improve lung cancer care (Table 3). We recommend adoption of the NCCN guidelines for lung cancer LDCT screening and prevention by physicians, health plans, medical groups, physicians, IPAs, HMOs, APMs and hospitals. To provide leadership, a clinical champion at the community site and also a champion at the academic center are important. The electronic medical record systems used should be updated to separately identify LDCT screening, prevention counseling and smoking cessation referrals and to enable lung cancer risk assessments. Compliance with guidelines should be measured, with reports back to physicians of their success rate or impact. High compliance should be promoted and rewarded. Additionally, the electronic health record can be better programmed to prompt or “nudge” health care providers to implement appropriate cancer screening [16]. Nudge interventions, such as automated alerts directed to the medical team to discuss breast cancer and colorectal cancer screening with patients, showed a dramatic increase in the number of ordered screening tests [17], but it did not affect how likely the patient was to actually complete the test within a year. Once cancer screening is ordered, the patient still has to take the right steps to complete it, such as scheduling an appointment and then going to the appointment. Hence, interventions should test methods to nudge both providers and patients to complete lung cancer screening, while also attempting to eliminate or mitigate some of the hurdles to these tests. 

Medical community and public promotion of lung cancer screening and prevention should be implemented. This will only be successful if each of the elements of screening and prevention are covered by insurance with reasonable copayments by patients. 

As science improves, there continues to be a need for clinical trials of the possibly beneficial improvements. These trials should be funded by government programs and/or voluntary health care agencies. As leaders in our communities, we should support these trials. 

## 6. Limitations of This Study

This study is at present observational and descriptive, and results will be determined prospectively with additional implementation of the initiative. The short follow up at present precludes detailed reporting of results on cessation success, reductions in lung cancer mortality, and cost savings. Results will be analyzed and published in future communications. Comparisons of lung cancer staging results with LDCT compared to staging resulting from diagnosis when symptoms were present will be evaluated when more patients have been diagnosed at Duarte and in the community network. 

## 7. Conclusions

Lung cancer continues to be a challenge to patients, providers, and health care systems despite dramatic improvements in treatment. We can use successful screening and prevention programs to reduce these life-threatening diagnoses and give patients more hope for the future. Smoking cessation in particular must become an integral component of cancer care and prevention. Reporting on the experience at City of Hope, the recommendations of the translational medical system of City of Hope, and the City of Hope Tobacco Control Initiative is designed to promote broader lung cancer screening and prevention efforts at other institutions and in other communities. We hope our experience will help contribute to improved patient outcomes and increase tobacco control. 

## Figures and Tables

**Table 1:** Sites for Lung Cancer Screening and Prevention.

Program	Academic Center	Affiliated Network Community Clinical Center	Collaborating Physicians and Hospitals
Screening Research	+++	+	_
Screening Implementation	+	++	+++
Prevention Research	+++	+	-
Prevention Implementation	+	++	+++
Provider Education	+++	+++	+
Patient Education	+++	+++	+++

**Table 2:** Factors in Translating Screening and Prevention Science from Academic Centers to Affiliated Network Community Centers and Collaborating Community Sites.

Factors Promoting Translation	Factors Limiting Translation
Establishing or accepting clear guidelines for screening and prevention	Insurers and IPAs desire reduced spending
Atmosphere of Collaboration and Congeniality	Contracts with physicians to reduce spending
Education at tumor boards and presentations	Limited resources at hospitals
Establishing screening and prevention programs at hospitals	Constricted time to focus on screening and prevention
Coordinating with voluntary health care organizations	Lack of payment for services or medications
American Cancer Society	Compliance surveys not focused on screening and prevention
American Lung Association	Lack of coordination
Cancer Support Community	High cost to patients
	Need to distinguish between screening for lung cancer versus diagnostic services to evaluate symptoms

**Table 3:** Recommendations for Improving Integration of Lung Cancer Screening and Prevention in Clinical Networks.

Recommendation	Responsibility of the Academic Center	Responsibility of Community Network Sites
Establish guidelines for ling cancer screening and prevention	+	
Identify clinical champions at the academic center and community site	+	+
Enhance electronic medical records to include screening LDCT and prevention counseling	+	+
Measure and reward compliance with guidelines	+	+
Promote screening with LDCT	+	+
Promote smoking cessation	+	+
Establish payment coverage for screening/prevention services	+	
Screening tests	+	
Prevention visits	+	
Counseling	+	
Smoking cessation program	+	
Smoking cessation drugs	+	
Fund prevention trials and fund medication trials for smoking cessation	+	

## List of Abbreviations

APM—alternative payment model organization;

APP—advanced practice provider;

COH—City of Hope;

COPD—chronic obstructive pulmonary disease;

HMO—health maintenance organization;

IPA—independent practice association;

IT—information technology;

LDCT—low-dose computed chest tomography;

L-RADS—lung imaging reporting and data system;

NCI—National Cancer Institute;

NCCN—National Comprehensive Cancer Network;

NRT—nicotine replacement therapy;

PPO—preferred provider organization;

Via—Via Oncology Clinical Pathways, now also known as ClinicalPath (Elsevier);
